# Diet and Gut Microbiome and the “Chicken or Egg” Problem

**DOI:** 10.3389/fnut.2021.828630

**Published:** 2022-02-01

**Authors:** Hannelore Daniel

**Affiliations:** Technical University of Munich, Munich, Germany

**Keywords:** intestine, microbiome, diet, determinants, treatment, diseases, transit time

## Abstract

Quantity and quality of the intestinal and fecal microbiome vary considerably between individuals and are dependent on a very large number of intrinsic and environmental factors. Currently, only around 15% of the variance in microbiome diversity can be explained by these factors. Although diet and individual food items have effects, other individual parameters such as gender, age, body mass index (BMI), but also plasma lipids and blood pressure reveal stronger associations with microbiome diversity. In addition, gastrointestinal functions that translate into changes in stool frequency, stool volume, and stool appearance rank very high as effectors of microbiome signatures. In particular, the intestinal/colonic transit time is a critical factor that alters the substrate load for bacterial growth and metabolism as it alters simultaneously stool volume, water content, bacterial mass, and diversity. Moreover, metabolic and neurological diseases are frequently associated with marked changes in intestinal transit time that may translate into the reported changes in gut microbiota. This review provides scientific arguments for a more comprehensive assessment of the individual's intestinal phenotype in microbiome studies to resolve the “chicken or egg” problem in these observational studies.

## Introduction

The interplay of the diet with the human gut microbiome has recently gained scientific and public popularity like no other aspect of food and nutrition sciences and there is hardly a disease that has not been linked to the composition of the gut microbiome. Since the diet is generally considered a key determinant of gut microbiome diversity, it is believed that dietary maneuvers easily alter the microbiota and thereby prevent diseases or slow disease progression. Targeted interventions to change the microbiome however that would require that we know what characterizes a “healthy microbiome.” But such a definition is still not available (1). Moreover, based on the huge variability in the composition of the microbiome across individuals but even within an individual with changes, day by day or depending on the time of sampling a “normal” microbiome is equally difficult to define and consequently “dysbiosis,” as deviation from normal, cannot be defined either (2). Yet, gut “dysbiosis” is often claimed as a critical factor in the susceptibility to and severity of diet-dependent diseases.

It appears as generally accepted that high bacterial diversity is the signature of a “healthy” microbiome although that is only based on observational evidence and is mainly derived from studies in which fecal samples from industrialized and nonindustrialized populations are compared. In addition to bacteria, the intestinal ecosystem harbors thousands of different viruses/bacteriophages (3) but also yeast and nematodes (see below). Although a huge number of variables have already been identified as contributing to microbiome diversity in populations from across the world and from different sociocultural and ecological environments those currently explain all-together only around 15% of the variance (1). It needs to be emphasized that almost all studies published in recent years provide only relative abundance data for the different phyla or genera of species in a sample. Given the fact that quantitative data are of utmost importance in all areas of biomedical and clinical research, the work with relative abundance data seems thus unique to microbiome science. Although various studies have assessed bacterial densities in the stool (4–6), a more recent analysis demonstrates that even a 10-fold difference in bacterial counts is not mirrored in relative abundance profiles (7). In addition, many variables affect the quality of analysis of stool samples (8) and the same sample analyzed by different laboratories can produce quite large differences in microbiome signatures (9). Given these caveats, caution should guide our recommendations to consumers interested in their microbiota. Here it will be critically assessed what influences bacterial density and diversity in stool samples and what diet effects have been observed in intervention studies. In addition, the question of whether the microbiome follows alterations in host physiology when moving into disease states or whether the microbiome is in a causative manner involved in disease initiation or progression will be discussed.

## Determinants of Human Gut Microbiome Diversity

Hundreds of population studies have meanwhile assessed microbiome profiles in stool samples and hundreds of parameters significantly associated with the diversity of microorganisms in the samples have been identified. Most interestingly, host genetics has only small effects with an inheritance of microbiota diversity accounting for around 2–9% (10, 11). As the most consistent finding in studies on host genetics and loci linked to the microbiome has the lactase gene been identified (12). Many other factors such as geographical origin or occupational state of the person rank also high among the key drivers of microbiome diversity.

Since stool samples from nonindustrialized communities but also paleosamples often display a wider range of bacterial species, these more diverse microbiomes are currently considered as a goal by dietary interventions. However, whether the highest diversity is the ultimate measure of a “healthy microbiome” has also been questioned (2). A very recent analysis of prehistoric stool samples (5,000–8,000 years old) from sites in the mid-west of the United States and Mexico (13) concluded that the bacterial diversity of these ancient samples is higher than that of samples representing industrial societies. However, the article reports as well that most paleosamples contained parasites. Another such example is stools of bronze-age miners recovered from salt mines in Austria with an exceptional preservation state and here also almost all samples contained eggs of various species of worms and other parasites (14). But even when modern samples from rural areas are analyzed, these frequently contain parasites and most interestingly, parasite infections associate with higher microbiome diversity (15, 16). Moreover, in animal studies, parasitic infections were shown to increase microbiome diversity which declined again when the infection was over (17). These observations ask whether high hygiene standards are also a critical determinant for less-diverse microbiomes.

**Figure 1:**
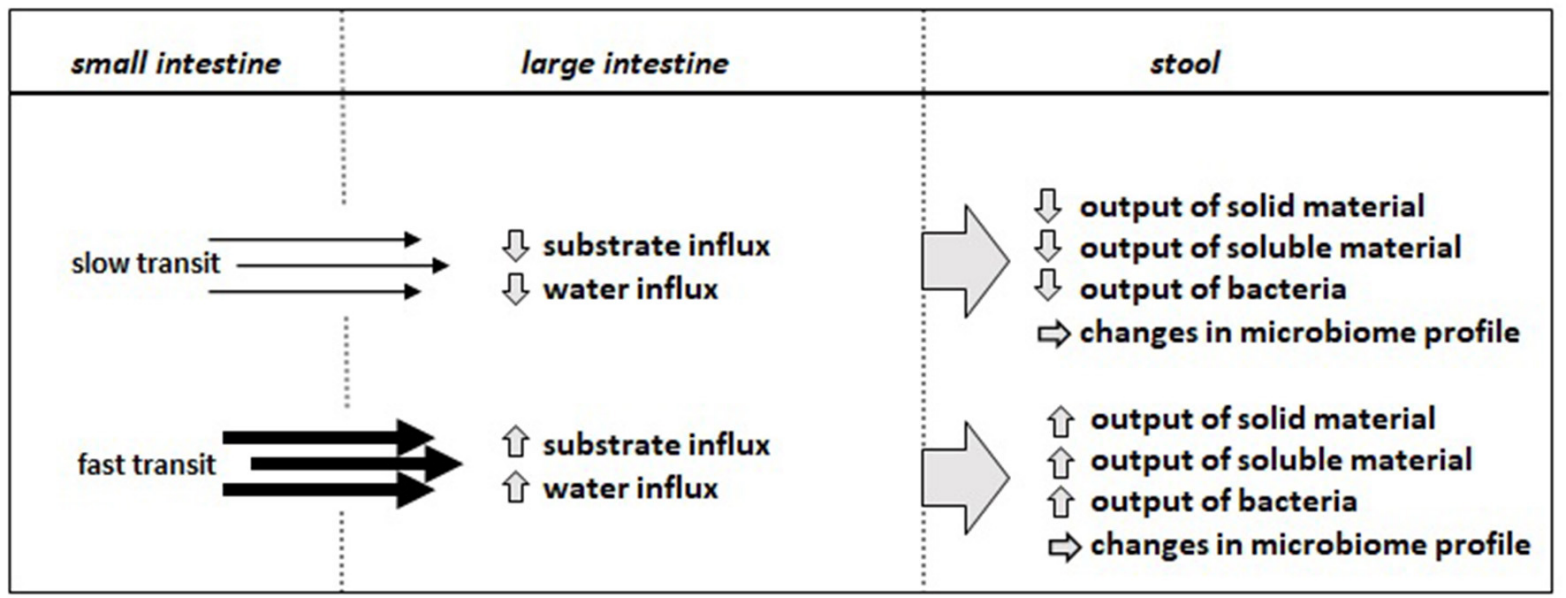
Parameters in fecal samples that change upon alterations of intestinal transit time based on findings derived from observational studies or intervention studies with agents that increase or slow-down transit time.

Among the factors that have the strongest association with stool microbiome diversity are age, sex, body mass index, and the Bristol Stool Scale (BSS) that classifies color and consistency of stool (18);( Falony, 2018). Although BSS can easily be defined based on a cartoon, it is unfortunately not often recorded. But stool water content has been related to microbiome diversity and the BSS contains the consistency of stool as a key classifier (19, 20). Stool water on the other hand is highly correlated with the gastrointestinal transit time (20) and the underlying motility program of the gut. Mean transit time (MTT) is highly variable and differs between men and women and is also age dependent (21, 22). Any maneuver that increases or decreases the gastrointestinal transit time causes changes in feces (Figure 1) with differences in bacterial density in stool samples and microbiome composition (23). Bacterial mass in the stool (g/day) can be altered almost 3-fold by MTT modifying agents and there is a close relationship between mass and the logMTT (24). In humans, colonic transit time measured by radio-opaque markers *via* x-ray correlates with the richness as diversity markers in stool samples (23). In a cohort of > 850 individuals in which transit time was measured, various species revealed a significant increase in relative abundance in stool with longer gut transit time while *Eubacterium rectale* density simultaneously decreased (25). The increased prevalence of the phylum *Bacteroidetes* with longer gut transit times had been observed before and similarly also an increase in *Akkermansia muciniphila* (20). In mice treated with loperamide to slow-down transit the density of *Bacteroidetes* was similarly observed (26). Along with those qualitative changes in stool bacterial signatures there are also changes in the bacterial counts in feces that increase by around 15% (per g dry weight) when transit is delayed or increase by nearly 20% when transit is increased (24). To which extent bacterial diversity is associated with the altered number of bacteria in stool samples is currently unclear.

Furthermore, MTT is a critical determinant for the rate of glucose absorption in the upper small intestine and the postprandial glucose profiles (27). By using a blue dye given in cupcakes for measuring transit time, it was shown to highly correlate with both, microbiome diversity and glycemic response to a carbohydrate load (25). These two read-outs appear to originate from a common intestinal phenotype—connected through motility and transit time with marked intraindividual differences. Interpretation of studies on postprandial glycemia in association with the microbiome should also take into account that high postprandial glucose concentrations (as found in individuals with insulin resistance or type 2 diabetes) can alter gastric, pancreatic, and intestinal responses to diet and change the transit time (28) and that these may well be factors contributing to microbiome changes reported in these disease states. A direct proof of the hypothesis that the MTT of the individual is a critical determinant of both, microbiome mass and diversity, and postprandial glucose responses requires further studies with a comprehensive analysis of all related parameters.

## Diet and Microbiome Composition

From population studies with the recording of food intake *via* 24-h recall or food frequency questionnaire, many food items have been identified as significantly contributing to microbiome diversity. That covers in essence almost all food and drink categories with very similar but generally very small effect sizes per item (18, 29). One of the most prominent factors that affect the microbiome in an unexpected manner is alcohol consumption (30) and which is prone to underreporting in observational studies. Yet, it confirms that food and drinks are all relevant factors among the many determinants of microbiome compositional signatures. However, not all studies provide convincing evidence that diet and microbiome diversity associate strongly. For example, volunteers consuming a chemically defined liquid diet as a meal replacement for 17 days did not show any significant difference in microbiome signatures (tested every day) when compared to volunteers consuming ordinary diets (31). Moreover, when volunteers consuming diets with average fiber content (mean fiber intake 22 g/day) were shifted to high fiber diets (mean fiber intake > 45 g/day) neither microbiome composition nor stool short-chain fatty acid (SCFA) concentration showed significant alterations (32). In a study in which volunteers consuming an entirely plant-based as compared to an animal-product based diet for 5 consecutive days with wash-out between the two arms, only in the animal-product-based arm, a significant effect on ß-diversity in stool was found (33). Intervention studies with fermentable fibers (12–15 g/day for 4 or even 12 weeks) as substrates for bacterial metabolism consistently report significant elevations in the abundance of a very few species, mainly of *Bifidobacteria* (8, 34), whereas overall microbiome diversity remained in almost all the studies unchanged [for review see also (35)]. Taken together, diet and many individual food items have been shown to associate with microbiome diversity, but intervention studies based on discrete diets or by providing dietary fibers of different quality and quantity so far provided only very limited evidence for successful steering of microbiota toward increased richness.

## Diet, Diseases, and Microbiome

Many noncommunicable diseases (NCDs) have been associated with sedentary lifestyles and dietary factors. In the Global Burden of Disease Studies, diet quality (intake of fruits, nuts, etc.) usually ranks high next to smoking, high blood pressure, BMI, and lack of physical activity (36). That now the microbiome and its diversity are brought into the health-disease trajectory is not surprising given the fact that microbiomes can easily be profiled these days for reasonable costs. Yet, the key question is of whether the microbiota is a causal factor in initiating or promoting diseases or whether changes in its composition just serve as a “reporter” of a disease state. The evidence that changes in the microbiome can affect disease severity or cause, is currently very limited. The best evidence may be provided by the outcomes of fecal microbiota transplantation (FMT) in which a suspension of feces from a healthy donor(s) is transmitted into a diseased person. This approach is the most successful treatment of recurrent *Clostridium difficile* infections (37) and is the “new gold” standard. Other attempts to alter disease progression or the physical state of the patient *via* FMT delivered less convincing or controversial outcomes (38). There are some trials in which treatment with FMT for people suffering from obesity, metabolic syndrome, or type 2 diabetes mellitus revealed some minor improvements but the more consistent finding was that there were no clinically significant effects (39, 40). Similarly, in patients with metabolic syndrome and elevated plasma trimethylamine-oxide (TMAO) levels, FMT with stool from a vegan donor was without effect on parameters of vascular inflammation (41). Epidemiological studies identified increased TMAO levels as associated with various cardiovascular disease types suggesting it to be a causative agent (42). TMAO is produced in the liver from trimethylamine produced in the microbiome from carnitine or choline and related compounds provided by the food of animal origin. Meanwhile, studies using *Mendelian Randomization Analysis* suggest that elevated blood TMAO levels may be an indicator of impaired renal clearance in these patients preselected for cardiovascular diseases rather than directly involved in pathogenesis (43). Taken together, changes in gut microbiota *via* stool transplantation have not yet convincingly demonstrated that metabolic health can significantly be improved. Similarly, treatment with antibiotics that caused severe alterations in microbiomes did also not significantly alter any of the markers of metabolic health in volunteers with type 2 diabetes (44). Thus, to establish that changes in microbiomes by any treatment has beneficial effects for metabolic health in NCDs, it needs larger trials with well-phenotyped volunteers or patients.

**Figure 2:**
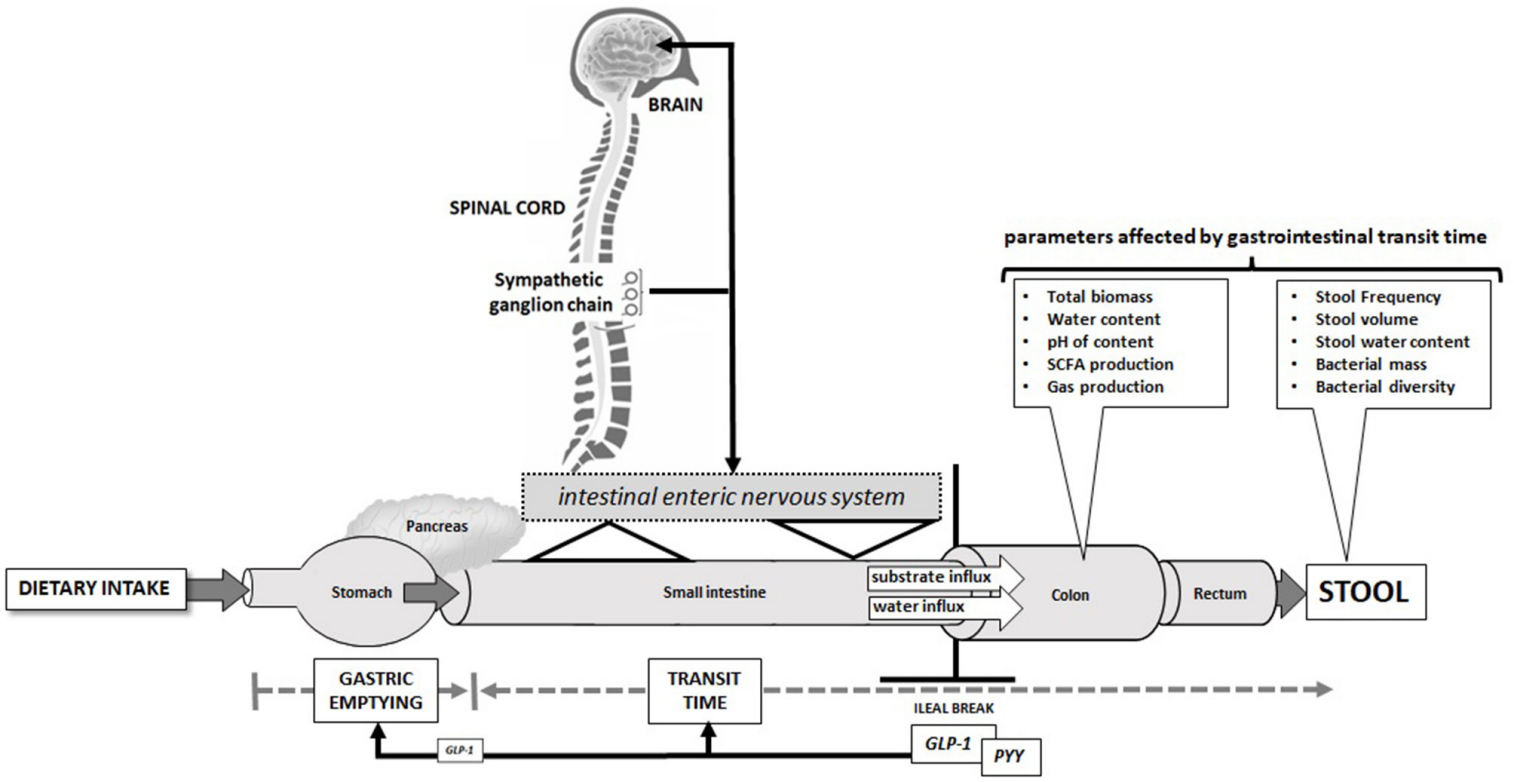
Selected physiological parameters that participate in the regulation of gastrointestinal motility and transit time and how alterations in transit time cause changes in the colonic microbiome and in stool characteristics that all have been demonstrated to alter microbiome diversity and biomass.

The intestine is a complex organ with an extensive neuronal network organized in plexi that receives multiple inputs from cells seeded into the mucosa from the stomach to the anus which are equipped with a multitude of sensors (45). The neuronal mesh underlying the mucosa acts in many ways as a mediator and is in contact with the sympathetic ganglion chain in the spinal cord and with the brain by which a bidirectional organ cross-talk of intestine and brain is realized (Figure 2). The intestine has in addition a large hormone system that produces numerous peptide hormones and a large number of amines, including the classical neurotransmitters, and those control in essence every process in the gut from digestion to absorption, to secretion and motility (46). Some of the hormones produced in enteroendocrine cells in the gut also reach peripheral organs and the brain and mediate satiety and metabolic control. Given these multidimensional networks connecting brain and gut, it is not surprising that many diseases—including neurological diseases—secondarily affect the gastrointestinal tract and its functionality.

The motility of the intestine translates into the surrogate of transit time and that is a critical determinant of the number of bacteria excreted with stool and of the diversity of the microbiome. Consequently, any alteration in transit time affects bacterial signatures (23, 24). Similarly, stool frequency has a significant effect on microbiome diversity (30, 47, 48) and a first genome-wide association study on stool frequency determinants has recently been published (49). When diseases have a demonstrated association with altered microbiome composition, it is thus important to assess whether intestinal functions are altered as the underlying cause of microbiome changes and that seems to be the case in many of the classical NCDs.

Obesity and metabolic syndrome have associated changes in gastrointestinal physiology with constipation, diarrhea, and fecal incontinence recognized as the most common intestinal complications in diabetes (50). They have their origin in changes in sensory functions with transmission into altered hormone and neuronal responses and changes in motor-neuron activities of the entire intestine (51). Other diseases with demonstrated alterations in microbiome profiles are Parkinson's disease (52) and Alzheimer's (53) disease, but also autism. A very recent meta-analysis of studies in Autism (54) reports that across various cohorts 45–85% of patients with autism have diarrhea or suffer from obstipation while a recent study in children with autism revealed that abnormal diet behaviors may be the prime reason for the changes in the microbiome and that the microbiota is not causing/promoting the disease (55). Thus, these neurological diseases have all associated changes in motility and motor function of the intestine and that is likely a major contributor to differences in bacterial density and diversity in stool samples (56, 57).

How are these changes in intestinal transit time changing the microbiota in stool? Alterations in transit cause changes in substrate flow across the ileocecal valve providing different substrate loads to the microbiota for utilization and growth. This influx of substrates into the colon is controlled *via* the “ileal break” which seems to sense the caloric load reaching the terminal ileum followed by the release of peptide hormones like glucagon-like peptide 1 or peptide YY that can reduce the gastric emptying rate and intestinal motility to allow better digestion/absorption (Figure 1). However, as shown by the use of compounds that change transit time in patients with ileostoma, different quantities of starch, for example, reach the colon when 50 g of potato starch are administered (58). Such maneuvers also change substantially the mean stool weight and bacterial mass in stool samples. A very interesting approach combined *in vivo* and *in-vitro* experiments (59) to assess the effects of transit time on fermentation of dietary fiber. It included the monitoring of SCFA levels and production rates and gas released when samples were collected from volunteers on identical diets but taking drugs that increase transit (cisapride) or slow transit (loperamide). Large differences in pH and SCFA concentration in the inoculum (stool) were already seen when transit time was altered, and fermentation *in vitro* also revealed major differences. A significant inverse relationship was found between SCFA production and the log of MTT, in analogy to previous studies that demonstrated such an inverse relationship also for the log MTT and the mean bacterial mass (g/day) excreted in the stool (24). That again demonstrates the close interrelationship of gut motility, colonic fermentation capacity, and the bacterial mass and spectrum in the large intestine and stool.

Taken together, there is convincing evidence that alterations in MTT occur in many diseases, and this alters the substrate load for fermentation in the colon including major effects on pH and SCFA concentrations associated with changes in stool frequency, stool volume/mass, stool water content, and, in turn, the amount and the composition of bacteria excreted. When inspecting all determinants of microbiome diversity identified so far, it appears that MTT is one of the most relevant factors and should therefore be determined in all studies that assess the links between diet, diseases, and microbiomes. There are various methods available to determine MTT with minimal efforts (60) including the use of colorants such as brilliant blue (E133) or spirulina to dye food items (25). Applying those methods would also help to overcome the “chicken or egg problem” in microbiome science.

## Author Contributions

The author confirms being the sole contributor of this work and has approved it for publication.

## Conflict of Interest

The author declares that the research was conducted in the absence of any commercial or financial relationships that could be construed as a potential conflict of interest.

## Publisher's Note

All claims expressed in this article are solely those of the authors and do not necessarily represent those of their affiliated organizations, or those of the publisher, the editors and the reviewers. Any product that may be evaluated in this article, or claim that may be made by its manufacturer, is not guaranteed or endorsed by the publisher.
